# Comparison of knowledge, perception and willingness to receive covid-19 vaccines among tertiary students in Osun State, Nigeria

**DOI:** 10.4314/ahs.v23i3.51

**Published:** 2023-09

**Authors:** Wale Adeyemi Johnson, Dauda Parakoyi Bayo

**Affiliations:** 1 Physiology Department, Adeleke University, Ede, Nigeria; 2 Community Medicine Department, Ladoke Akintola University of Technology (LAUTECH), Ogbomoso, Nigeria

**Keywords:** Covid-19 vaccines, knowledge, perception, tertiary students, willingness

## Abstract

**Background:**

Vaccination remains a potent way to curb the present covid-19 global pandemic

**Objectives:**

To assess knowledge, perception and willingness to receive covid-19 vaccines among tertiary students in Nigeria.

**Methods:**

In the descriptive cross-sectional study, a sample size of 750 respondents was randomly selected from a university, polytechnic and college of education (COE) in Osun State, Nigeria. Independent sample T and Pearson correlation tests were used to analyse the responses.

**Results:**

There was a significant increase in the percentage score of poor perception, relative to good perception among the university and polytechnic respondents. Among the COE respondents, significant increases in the percentage scores of poor knowledge, perception and willingness to receive covid-19 vaccines, relative to the good variables were observed. Weak positive correlations between knowledge and willingness & perception and willingness to receive covid-19 vaccines among all the respondents were noted. In addition, there was a significant increase in good perception to covid-19 vaccines among university and COE, relative to polytechnic respondents. Asides, a significant increase in good willingness to receive covid-19 vaccines was observed among the university, compared to COE respondents.

**Conclusion:**

There is poor knowledge, perception and willingness to receive covid-19 vaccines among tertiary students in Osun State, Nigeria.

## Introduction

Coronavirus pandemic has become a threat to the global economic and health systems. The daily increases in the number of infected persons and death have resulted in the emergence of various means to save the masses. Preventive interventions like the use of face mask/shield, hand washing and sanitising, social distancing and avoidance of crowded places, and coughing and sneezing into the handkerchief or elbow, among others, have played a major role in reducing causalities. However, these precautionary measures cannot guarantee a total stoppage in the spread of the virus. Therefore, efforts were committed towards the development of vaccines, which includes Johnson and Johnson, Pfizer-BioNTech, Moderna/NIAID, and Oxford-AstraZeneca^1^, with the prospects of finding a lasting solution to this common problem.

Fair and effective distribution of covid-19 vaccine has been emphasised. Equally important is the acceptance of the vaccine by the people. The success of any vaccination campaign depends on trust in the vaccines and the institutions through which it is administered^2^. There are several reports in literature on the willingness of residents in high- and middle- income countries to covid-19 vaccines^3^-^5^. However, there is dearth of information on the acceptance of the vaccine in a low-income country like Nigeria, where large scale vaccination is yet to begin, and yet with lots of negative speculations and controversies about the real intent of the vaccines. The acceptance of covid-19 vaccine by everyone everywhere cannot be undermined especially because a deficit in the efforts of any country towards vaccination may result in the emergence and spread of a new strain of the virus that can escape the immunity offered by the administered vaccines^6^, causing more morbidities and mortalities.

Although the covid-19 vaccines that are being administered have been reported to be safe; nevertheless, there are associated mild symptoms and sometimes fatalities, 7 which is no doubt cause anxiety and fear 8. Other notable factors that have been reported to influence the disposition of the general population about the acceptance of vaccines include inadequate knowledge about the potency and long-term effects, demographic characteristics, perceptive influences, and lack of trust in the health care system^9^,^10^. Several studies have noted hesitancy and refusal of vaccines in general and covid-19 vaccines in particular, among people in different countries 11-14. A recent review opined that vaccination rejection rates in the world varied widely from as low as 4.3% to as high as 72% 15.

Presently, no vaccines have been developed in Africa, yet, little clinical trials have been conducted in this part of the world^16^. These seemed to have inflated the resistance of the people towards the vaccine, coupled with the frequent racist attack in several parts of the world. The seemingly low acceptance rate of covid-19 vaccine in Nigeria^17^ needs to be investigated among the teaming youths, majority of which are in the higher institutions of learning. Tertiary institutions in Nigeria are categorised into three levels – universities, polytechnics, and colleges of education. The universities top the list, while the colleges of education are considered to be the least. Hence, it is important to assess educated individuals across different tertiary institutions on the developed covid-19 vaccines, especially because the few reports in literature on the knowledge, perception and willingness were not focused on this group^18^-^20^. Therefore, the present study was aimed at assessing the knowledge, perception and willingness of randomly selected university, polytechnic and college of education students in Osun State, Nigeria to covid-19 vaccine.

## Methods

### Ethical consideration

Ethical approval for the study was obtained from the Research and Ethics Committee of the College of Health Sciences, Ladoke Akintola University of Technology (LAUTECH), Ogbomoso, Nigeria. Verbal and/or written informed consents were sought from each respondent after a brief discuss on the objectives of the study. Ethical approval number was not assigned for this study.

### Study area, population and design

This descriptive cross-sectional study was carried out in Osun State, located in the south-west of Nigeria. A tertiary institution was selected among the three categories of higher institutions (i.e., university, polytechnic and college of education) in Osun state. Simple random sampling technique was used to select the following institutions, viz; a university (Redeemer's University, Ede), a polytechnic (Federal Polytechnic, Ede), and a college of education (Osun State College of education, Ilesha). Data were collected over two weeks in the month of June, 2021. The estimate population of the three institutions is about three thousand.

A pre-tested 5-point Likert scale questionnaire was used to collect the data of the respondents, which included the socio-demographic characteristics and responses on knowledge, perception and willingness to receive COVID-19 vaccines. The questionnaire was randomly distributed among a total of 750 respondents, which included second year students studying medical-related courses e.g., Anatomy, Physiology, Nursing, Physiotherapy, etc and those studying non-medical related courses e.g., Engineering, Life Sciences, Agriculture Faculties etc). Two fifty (250) respondents were targeted in each of the three selected institutions.

### Sample size determination

Sample size was determined using the formula – N = Z^2^ P (1-P)/d^2^ at a confidence level of 95%, unknown proportion (P) of 50% and 6% error margin (d).

### Data collection

The questionnaire that was administered consisted of four sections. Section A contained questions on the socio-demographic characteristics of the respondents, while section B, C, and D assessed the knowledge, perception, and willingness of the respondents to COVID-19 vaccines. The sections B-D were rated on a 5-point Likert scale, where 1 represented “Absolutely No”, 2 “No”, 3 “Not sure”, 4 “Yes” and 5 “Absolutely yes.”

The mean and percentage score of poor knowledge, perception and willingness were derived from the respondents that ticked absolutely no, no, and not sure, while the mean and percentage score of good knowledge, perception and willingness were derived from the respondents that ticked yes and absolutely yes.

### Data processing and analysis

Data obtained from the study were analysed using SPSS version 20 for windows (IBM SPSS Inc., Chicago, USA). Descriptive statistics were reported in frequencies and percentages and independent sample T test was used to compare the mean values of knowledge, perception and willingness of the respondents. While, Pearson correlation analysis was used to determine the strength of the association between the mean values of the responses. *P*<0.05 was considered to be statistically significance.

## Results

### The demographics data of the 750 respondents at the university, polytechnic and college of education

Table 1a shows the demographics data of the 750 respondents at the university, polytechnic and college of education. Data on age, sex, religion, course of study, covid-19 vaccine status, covid-19 status, political party and social class were presented in counts and percentages.

**Table 1a:** The demographics data of 750 respondents randomly sampled among tertiary students in Osun State, Nigeria

		University	Polytechnic	College of Education
S/No	Characteristics	N (%)	N (%)	N (%)
Age	<18	64 (25.6)	53 (21.1)	50 (20.0)
	>18	186 (74.4)	197 (78.9)	200 (80.0)
Sex	Female	172 (68.9)	147 (58.9)	156 (62.2)
	Male	78 (31.1)	103 (41.1)	94 (37.8)
Religion	Islam	6 (2.2)	97 (38.9)	69 (27.8)
	Christian	244 (97.8)	153 (61.1)	181 (72.2)
Course of study	Non-medical related course	117 (46.7)	136 (54.4)	97 (38.9)
	Medical related course	133 (53.3)	114 (45.6)	153 (61.1)
Covid-19 vaccines status	Non-vaccinated	231 (92.2)	233 (93.3)	233 (93.3)
	Vaccinated	19 (7.8)	17 (6.7)	17 (6.7)
Covid-19 status	Negative	203 (81.1)	103 (41.1)	112 (44.4)
	Positive	3 (1.1)	0 (0)	22 (8.9)
	Not sure	44 (17.8)	147 (58.9)	116 (46.7)
Political party	PDP	69 (27.8)	72 (28.9)	114 (45.6)
	APC	78 (31.1)	61 (24.4)	69 (27.8)
	Other parties	103 (41.1)	117 (46.7)	67 (26.7)
Social class	Lower	0 (0)	22 (8.9)	14 (5.6)
	Middle	164 (65.6)	170 (67.8)	142 (56.7)
	Upper	86 (34.4)	58 (23.3)	94 (37.8)

### Mean and standard deviation on assessment of knowledge, perception and willingness to receive covid-19 vaccines among tertiary students in Osun State, Nigeria

The table 1b shows the mean and standard deviation values on the assessment of knowledge, perception and willingness to receive covid-19 vaccines among university, polytechnic and college of education respondents. The lowest values of 2.41±1.10, 2.42±1.41, 2.88±1.39 and highest values of 4.22±4.48, 3.58±1.18, 3.27±1.36 on the assessment of knowledge of the respondents about covid-19 vaccine were recorded among the university, polytechnics and college of education respondents, respectively (Table 1bi). Moreover, lowest values of 2.23±1.41, 1.89±1.19, 2.58±1.39 and highest values of 3.46±4.61, 2.93±1.39, 3.13±1.24 on the assessment of perception of the respondents about covid-19 vaccine were recorded among the university, polytechnics and college of education respondents, respectively (Table 1bii). In addition, lowest values of 2.63±0.92, 2.49±1.07, 2.96±1.25 and highest values of 3.56±1.14, 3.36±1.34, 3.24±1.28 on the assessment of willingness of the respondents to receive covid-19 vaccines were recorded among the university, polytechnics and college of education respondents, respectively (Table 1biii).

**Table 1b:** Mean and standard deviation on assessment of knowledge, perception and willingness to receive covid-19 vaccines among tertiary students in Osun State, Nigeria

	Table 1bi	University	Polytechnic	College of education
S/No	Assessment on knowledge on covid-19 vaccines	Mean ± SD	Mean ± SD	Mean ± SD
1	Protection of people from the corona virus by covid19 vaccines	3.49±1.34	3.58±1.18	3.27±1.36
2	Availability of covid-19 vaccines in Nigeria	3.10±1.43	3.18±1.34	3.22±1.30
3	Availability of covid-19 vaccines for selected persons	2.41±1.10	2.57±1.02	3.06±1.14
4	Necessity of everyone to get covid-19 vaccines	3.23±1.32	3.33±1.35	3.06±1.28
5	Prioritization of health professionals in covid-19 vaccines administration	4.22±4.48	3.16±1.39	3.07±1.33
6	Vaccination of pregnant women	2.57±1.45	3.03±1.46	2.92±1.39
7	Vaccination of lactating women	2.57±1.43	2.74±1.42	3.08±1.43
8	Prioritization of the elderly in covid-19 vaccines administration	3.61±1.15	3.07±1.49	2.88±1.39
9	Reduction of the prevalence of coronavirus disease after introduction of covid-19 vaccines	2.78±1.50	2.42±1.41	2.89±1.32
10	Possibility of fully vaccinated person to contact the virus	2.67±1.44	2.30±1.27	2.99±1.38

	Table 1bii	University	Polytechnic	College of education
S/No	Assessment of perception on covid-19 vaccines	Mean ± SD	Mean ± SD	Mean ± SD
1	Perception on effectiveness of covid-19 vaccines compared to hepatitis, polio, measles and tuberculosis vaccines	2.23±1.41	2.21±1.44	2.58±1.39
2	Perception on stigmatisation of those that received covid-19 vaccines	2.61±0.92	2.40±1.26	2.78±1.29
3	Perception on whether those that died as a result of coronavirus would be alive if they had taken covid-19 vaccines	2.38±1.35	2.60±1.52	2.66±1.36
4	Perception on whether covid-19 vaccines is safe	2.59±1.47	2.93±1.39	3.02±1.31
5	Perception on whether covid-19 vaccines can alter your genetic make-up	2.26±1.20	1.89±1.19	2.76±1.27
6	Perception on whether covid-19 vaccines were aimed at wiping out the African race	2.46±0.95	2.19±1.24	2.91±1.35
7	Perception on whether one needs to be connected before one can get covid-19 vaccines	2.60±1.14	2.76±1.15	3.02±1.22
8	Perception on whether the covid-19 vaccines given to the elites/prominent people are the same as that given to the common people	2.64±1.38	2.30±1.37	2.71±1.29
9	Perception on whether covid-19 vaccines can adversely affect academic performance	3.46±4.61	2.60±1.47	3.13±1.24
10	Perception on whether covid-19 vaccines is a mark of the Devil	2.52±0.93	2.31±1.25	3.04±1.23

	Table 1biii	University	Polytechnic	College of education
S/No	Assessment of willingness to receive covid-19 vaccines	Mean ± SD	Mean ± SD	Mean ± SD
1	Willingness to lose your studentship rather than receive covid-19 vaccines if the management of your institution made it compulsory	2.63±0.92	2.49±1.07	3.00±1.20
2	Willingness to receive covid-19 vaccines even if your religious leaders disapprove it	2.97±1.25	2.57±1.32	3.02±1.26
3	Willingness to receive covid-19 vaccines if you are sure that the vaccines given the Nigerian President will be given to you	2.94±1.22	2.58±1.32	2.98±1.25
4	Willingness to receive covid-19 vaccines if your parents demanded that you should take it	3.56±1.14	3.09±1.44	3.24±1.28
5	Willingness to receive covid-19 vaccines if there are no reports in the news about its adverse effects	3.18±1.27	2.63±1.29	2.96±1.25
6	Willingness to receive covid-19 vaccines if you are given assurance by your family doctor that all will be well	3.09±1.27	3.02±1.41	3.07±1.31
7	Willingness to receive covid-19 vaccines if you noticed that you are showing symptoms of coronavirus infection	3.24±1.35	3.36±1.34	3.04±1.26
8	Willingness to receive covid-19 vaccines if it is made available at your door step	2.82±1.38	2.64±1.44	3.09±1.15
9	Willingness to receive covid-19 vaccines prior to national youth service corps (NYSC) mobilisation, knowing fully-well that you will interact with people from all over the country	3.22±1.40	2.98±1.42	3.16±1.16
10	Willingness to receive covid-19 vaccines if you have to relocate to a high-risk zone	3.28±1.28	2.84±1.40	3.03±1.18

### Knowledge, perception and willingness to receive covid-19 vaccines among tertiary students in Osun State, Nigeria

The tables 2 a-c show the data obtained on the assessment of knowledge, perception and willingness to receive covid-19 vaccines among respondents at different levels of tertiary institution. The table 2a shows that an insignificant difference of p - 0.368 and 0.750 were recorded between the mean scores of poor and good knowledge & poor and good willingness respectively among university respondents. However, there was a significant increase (p - 0.011) in poor perception, relative to good perception among this population. The table 2b reveals that insignificant differences of p - 0.169 and 0.135 were recorded between the mean scores of poor and good knowledge & poor and good willingness respectively among the respondents. However, there was a significant increase (p - 0.011) in poor perception, relative to good perception among this population. The table 2c shows that there were significant increases (p - 0.000, 0.000, 0.000) in poor knowledge, poor perception, and poor willingness, relative to good variables among this population.

**Table 2:** Poor and good knowledge, perception and willingness to receive covid-19 vaccines among tertiary students in Osun State, Nigeria

Features	Mean ± SEM	Percentage (%)	*P* - value
**2a. University** **respondents**			

Poor knowledge	41.80±17.66	46.4	0.368
Good knowledge	48.20±18.05	53.6	
Poor perception	51.35±15.88	57.1	0.011
Good perception	38.65±15.96	42.9	
Poor willingness	42.80±11.61	47.6	0.540
Good willingness	47.20±11.55	52.4	

**2b. Polytechnic** **respondents**			

Poor knowledge	48.25±16.57	53.6	0.169
Good knowledge	41.75±16.36	46.4	
Poor perception	62.20±8.81	69.1	0.000
Good perception	27.80±8.82	30.9	
Poor willingness	47.65±11.75	52.9	0.135
Good willingness	42.35±11.65	47.1	

**2c. College of Education respondents**			

Poor knowledge	49.65±6.25	55.17	0.000
Good knowledge	40.35±5.99	44.83	
Poor perception	50.00±5.79	55.56	0.000
Good perception	40.00±6.08	44.44	
Poor willingness	49.50±5.42	55.00	0.000
Good willingness	40.50±5.44	45.00	

*p* < 0.05 is considered to be significant

### Correlations between knowledge, perception and willingness to receive covid-19 vaccines among tertiary students in Osun State, Nigeria

Table 3 shows that when comparison was made among knowledge, perception and willingness to receive covid-19 vaccines among the university, polytechnic and college of education respondents. There were weak positive correlations of 0.127 and 0.123 between knowledge & willingness and perception & willingness to receive covid-19 vaccines respectively among the university respondents. The table 3 further shows the weak positive correlations of 0.335 and 0.232 that were noted between knowledge & willingness and perception & willingness respectively among the polytechnic respondents. However, a weak and a fairly strong positive correlations values of 0.254 and 0.622 were observed between knowledge & willingness and perception & willingness respectively to covid-19 vaccines among the college of education respondents.

**Table 3:** Correlations between knowledge, perception and willingness to receive covid-19 vaccines among tertiary students in Osun State, Nigeria

Characteristics	University	Polytechnic	College of Education
Knowledge *vs* willingness	0.127	0.335	0.254
Perception *vs* willingness	0.123	0.232	0.622

### Significance difference between the mean value of good and poor knowledge, perception and willingness to receive covid-19 vaccines among tertiary students in Osun State, Nigeria

The table 4 shows that there were significant increases in poor perception about covid-19 vaccines among polytechnic respondents, relative to university and college of education (*p –* 0.012, 0.000 respectively). Otherwise, there were significant increases in good perception about covid-19 vaccines among university and college of education, compared to polytechnic respondents (*p –* 0.014, 0.000 respectively). A significant decrease (*p –* 0.027) in poor willingness to receive covid-19 vaccines was noted among university respondents, relative to college of education. On the other hand, there was a significant increase *(p* - 0.023) in good willingness to receive covid-19 vaccines among university respondents, relative to college of education respondents.

**Table 4:** Significance difference between the mean value of good and poor knowledge, perception and willingness to covid-19 vaccines among tertiary students in Osun State, Nigeria

Comparison	Poor knowledge	Good Knowledge	Poor perception	Good Perception	Poor willingness	Good willingness
University	41.80±17.66	48.20±18.05	51.35±15.88	38.65±15.96	42.80±11.61	47.20±11.55
vs	*vs*	*vs*	*vs*	*vs*	*vs*	*vs*
Polytechnic	48.25±16.57	41.75±16.36	62.20±8.81*p*-	27.80±8.82	47.65±11.75	42.35±11.65
	*p*-0.241	*p*-0.278	0.012	*p*-0.014	*p*-0.197	*p*-0.190
University	41.80±17.66	48.20±18.05	51.35±15.88	38.65±15.96	42.80±11.61	47.20±11.55
vs	*vs*	*vs*	*vs*	*vs*	*vs*	*vs*
College of Education	49.65±6.25	40.35±5.99	50.00±5.79	40.00±6.08	49.50±5.42	40.50±5.44
	*p* -0.073	*p* -0.089	*p* -0.724	*p* -0.856	*p* -0.027	*p* -0.023
Polytechnic	48.25±16.57	41.75±16.36	62.20±8.81	27.80±8.82	47.65±11.75	42.35±11.65
*vs*	*vs*	*vs*	*vs*	*vs*	*vs*	*vs*
College of	49.65±6.25	40.35±5.99	50.00±5.79	40.00±6.08	49.50±5.42	40.50±5.44
Education	*p* -0.727	*p* -0.694	*p* -0.000	*p* -0.000	*p* -0.528	*p* -0.493

*p* < 0.05 is considered to be significant

## Discussion

The emergence of various strains of coronavirus has not only drained the economy of various nations, but also the health sectors. With the recent shocking of the omicron strain of the virus and the commencement of the administration of booster doses of covid-19 vaccines, it has become clear that vaccination remains a potent way and probably the last resort to rescue the global populace from the devastating effects of covid-19 pandemic. This is evidence from the development of various vaccines by different multinational pharmaceutical companies and research organisations. However, the effectiveness of this preventive measure hinges on the knowledge, perception and willingness of individuals to receive the available vaccines, especially in Africa, where there were limited clinical trials, and manufactured vaccines.

This study is important because a deficit in vaccination in any country might lead to the emergence and spread of a new strain of the virus that can overcome protection conferred by vaccines^6^. For the first time, we examined knowledge, perception and willingness to receive receive covid-19 vaccines among students in different categories of tertiary institutions – college of education, polytechnic and university, in Osun State, Nigeria. Ezeani *et al.*^18^ noted that social media, television/radio and internet are the three major sources of information about the vaccines. Moreover, they recorded a significant level of knowledge about the vaccines among Akwa residents (educated and uneducated) in Anambra State. In contrast to their report, in this study, we observed insignificant differences in and good knowledge about covid-19 vaccines among the university and polytechnic respondents.

This could be attributed partly to the high tasking questions used in this study, which was considered to be within the reach of educated individuals. Although, there was substantial awareness about covid-19 vaccines in various communication media, the insignificant increase in good knowledge about the vaccines across the three institutions (table 2a-c) could also be attributed to erratic power supply in the country, which could have prevented the students from access to some information especially on the television and radio. Moreover, tight schedule of the students with school activities might have robbed them from other information sources outside their courses. This is especially because the academic calendar of most institutions was compressed in order to make up for lapses of the students during the lockdown. This has indeed created more academic pressure for the students, and so majority seems not to be interested in anything that is not directly related to their studies.

The result is poor knowledge and perception about covid-19 vaccines. Comparatively, there were no significant differences in the level of knowledge about covid-19 vaccines among respondents in the three institutions. Hence, it would not be out of place if information about covid-19 virus and the vaccines is directly incorporated into the study context of the students. Anorue and colleagues corroborated the findings of this study on the low level of knowledge among Nigerian, with a focus on the south-east residents. The researchers submitted that knowledge of respondents about the safety of the vaccines is low and has resulted in respondents' attitude towards it.

Generally, knowledge is thought to influence perception 21 and perception in turn influences readiness and willingness attitude 22. The results of this study shows that significant percentages of the respondents in the three institutions have a poor perception about covid-19 vaccines - 57.1%, 69.1% and 55.56% at the university, polytechnic and college of education respectively. The insignificant level of good knowledge about the vaccines translated to the observed poor perception and hence the consequent unwillingness among the respondents to receive the vaccines. Comparatively, there was a significant increase in good perception to covid-19 vaccines among university and COE, relative to polytechnic respondents. The reason for this could not be ascertained.

The unwillingness of the respondents towards covid-19 vaccines seems to be peculiar to the vaccines in particular and not any other type of vaccines^18^. It was not expected that insignificant increase in good knowledge and perception about covid-19 vaccines noted in this study would translate to willingness to receive the vaccines. Moreover, a significant high level of good knowledge and perception about the vaccines may not necessarily results in high level of willingness to receive the vaccines among the respondents, as reported by Agbo *et al.* 17 The result of this study shows that there were no significant differences in poor and good willingness to receive covid-19 vaccines among the university and polytechnic respondents. However, a significant proportion of the respondents at the college of education showed unwillingness (poor willingness) to receive the covid-19 vaccines. The generalised unwillingness of the respondents to the vaccines was demonstrated in the demographic data, which revealed that a large percentage of the respondents at the university (92.2%), polytechnic (93.3%) and college of education (93.3%) have not yet been vaccinated. In unison with this study, Josiah *et al.* 19 and Ekwebene *et al.* 23 reported a low level of willingness to receive covid-19 vaccines among the Nigerian populace, with focus on Delta state residents and Healthcare Providers in the six geopolitical zones of the country, respectively. The surprisingly unwillingness to receive covid-19 vaccines could be partly attributed to fear of possible adverse effects, false perception that Nigerians are naturally immune to the virus, false perception that the infection affects only the rich and influential, thoughts that covid-19 infection is a scam, and that the vaccines was aimed at population reduction and microchip implantation. Moreover, the low level of trust of the general populace in the government, partly due to the economy instability, high level of inflation, growing insecurity and unfulfilled election campaign promises, among others, seems to have contributed to the lack of trust of the residents in every other programme and agenda of the present administration. Trust is very crucial for vaccines acceptance, and high rate of compliance and uptake 20.

Comparatively, there was a significant increase in good willingness to receive covid-19 vaccines among the university respondents, compared to COE respondents. This could be ascribed to the fact that university students are considered to have a broader based-knowledge than those at the college of education. Ankrah and Nwaigwe 24 demonstrated that religion play a major role in vaccination programme. They indicated that Christians are more positively disposed to vaccination compared to the Muslims. In this study, most of the respondents are Christians – 78%, 61.1% and 71.2% in the university, polytechnic and college of education, respectively, yet majority of the respondents across the three institutions showed unwillingness to receive the vaccines. Hence, the role of religion on willingness to receive vaccination needs to be reconsidered. It is noteworthy, that most religious leaders in Nigeria seemed to be negatively disposed towards the vaccines, at least when it was first introduced. In fact, the vaccines have been erroneously associated with the mark of the beast – 666 25. Therefore, a regular Christians in most denomination were expected to conquer the virus by faith, by that it is believed that they are following after their father in the Lord. Hence, government needs to partner with various religious organisations if the country would ever attain the desired level of acceptance of vaccination among the general populace.

Interestingly, we noted significant higher levels of poor knowledge, perception and willingness to receive covid-19 vaccines, relative to the good variables, among the respondents in the college of education in particular. In the Nigerian education system, the college of education is considered to be the lowest level of the higher educational system. We may not be able to directly attribute the observed result to this fact; however, it could be implicated. Most importantly, this result shows that there should be a higher level of sensitisation about covid-19 vaccines among students at the college of education, compared to those at the polytechnics and the universities. The weak correlation between knowledge and willingness & perception and willingness among the respondents to covid-19 vaccines in each institution indicated that the poor knowledge and perception about covid-19 vaccines were the direct consequence of the unwillingness of the respondents to take the vaccines. Majority of the respondents were not yet vaccinated even though vaccines are available in various primary health care centres in Osun State, Nigeria and in the Redeemer's university health centre in particular.

## Conclusion

There is poor knowledge, perception and willingness to receive covid-19 vaccines among tertiary students in Osun State, Nigeria, most especially among the COE and polytechnic students; hence, there is need for adequate sensitisation.
